# Supplementary material for: Potential of the Stromal Matricellular Protein Periostin as a Biomarker to Improve Risk Assessment in Prostate Cancer
Source: Int J Mol Sci. 2022 Jul 20;23(14):7987. doi: 10.3390/ijms23147987 (PMC9324424; doi:10.3390/ijms23147987)
Supplement: Supplementary file 1 [file ijms-23-07987-s001.zip › Supplementary Figure S1.pdf]

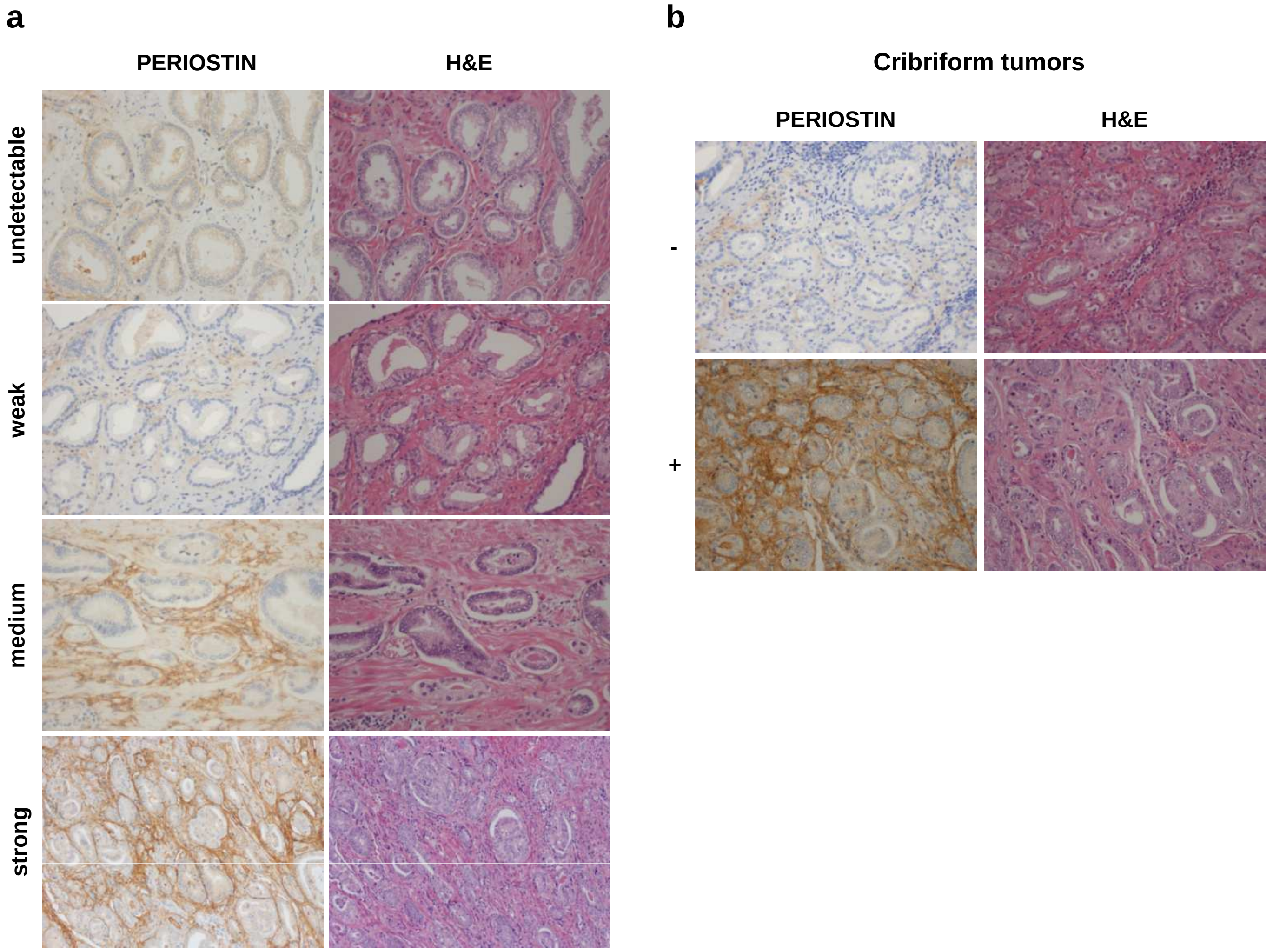

Supplementary Figure S1

Periostin staining in PCa tissue section and relative and hematoxylin and eosin (H&E) staining for morphological evaluation. **(a)** Full representative images of undetectable, weak, medium and strong periostin staining and H&E. Magnification: 200x. **(b)** Full representative images periostin staining and relative H&E in GS 4 PCa tumors with or without cribriform morphology. Magnification: 200x.

Supplementary figure S1
