# Supplementary material for: Potential of the Stromal Matricellular Protein Periostin as a Biomarker to Improve Risk Assessment in Prostate Cancer
Source: Int J Mol Sci. 2022 Jul 20;23(14):7987. doi: 10.3390/ijms23147987 (PMC9324424; doi:10.3390/ijms23147987)
Supplement: Supplementary file 1 [file ijms-23-07987-s001.zip › Supplementary Figure S2.pdf]

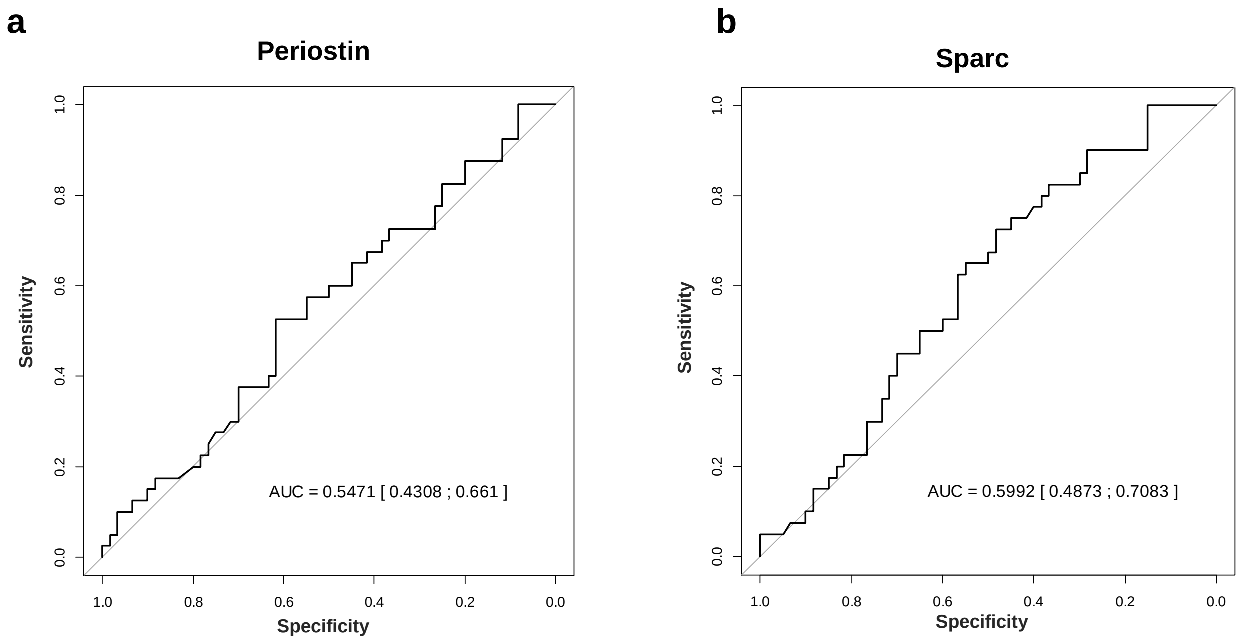

ROC curve of circulating periostin and sparc in AS patients. (a) ROC curve of circulating periostin in 100 AS patients. (b) ROC curve of circulating sparc in 100 AS patients.

Supplementary figure S2
