# Supplementary material for: Potential of the Stromal Matricellular Protein Periostin as a Biomarker to Improve Risk Assessment in Prostate Cancer
Source: Int J Mol Sci. 2022 Jul 20;23(14):7987. doi: 10.3390/ijms23147987 (PMC9324424; doi:10.3390/ijms23147987)
Supplement: Supplementary file 1 [file ijms-23-07987-s001.zip › Supplementary Figure S3.pdf]

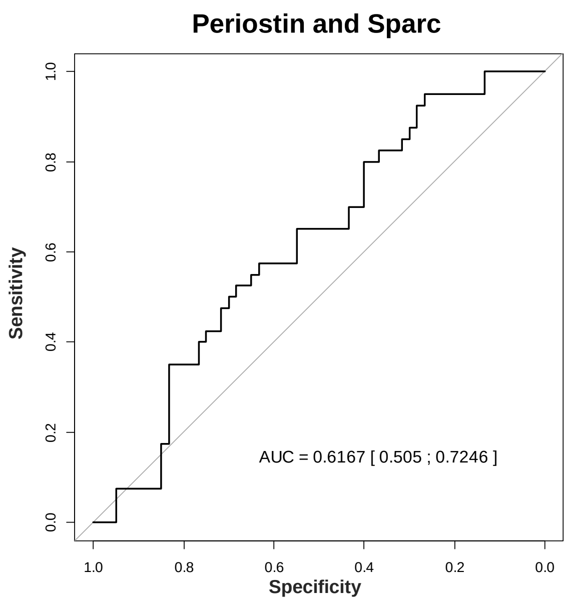

Logistic model including circulating periostin and sparc in AS patients. ROC curve of the logistic model including circulating sparc and periostin in 100 AS patients.

Supplementary figure S3
