# Supplementary material for: Potential of the Stromal Matricellular Protein Periostin as a Biomarker to Improve Risk Assessment in Prostate Cancer
Source: Int J Mol Sci. 2022 Jul 20;23(14):7987. doi: 10.3390/ijms23147987 (PMC9324424; doi:10.3390/ijms23147987)
Supplement: Supplementary file 1 [file ijms-23-07987-s001.zip › Supplementary Table S1.pdf]

Supplementary Table S1. Clinico-pathological characteristics of PCa patients who underwent radical prostatectomy.

**Variable**		**n**	**%**
Gleason score			
	3+3	38	32.76
	3+4	40	34.48
	3+5	3	2.59
	4+3	20	17.24
	4+4	11	9.48
	4+5	3	2.59
	5+3	1	0.86
PGG			
	1	38	32.76
	2	40	34.48
	>2	38	32.76
Cribriform morphology			
	no	58	50
	yes	16 (14*)	13.79 (12.06*)
	undef	42	36.21
Resection margins			
	0	78	67.24
	1-2	34	29.31
	.	4	3.45
Seminal vesicle invasion			
	0	100	86.21
	1	12	10.34
	.	4	3.45
EPE			
	no	71	61.21
	yes	41	35.34
	.	4	3.45
Periostin			
	0-1	47	40.52
	2-3	69	59.48

*Periostin: periostin stroma staining; PGG: prognostic grade group; EPE: extraprostatic extension. * sub-cohort of GS=3+4/4+3 (PGG2/3) tumors analyzed in figure 2C.*
