# Supplementary material for: Potential of the Stromal Matricellular Protein Periostin as a Biomarker to Improve Risk Assessment in Prostate Cancer
Source: Int J Mol Sci. 2022 Jul 20;23(14):7987. doi: 10.3390/ijms23147987 (PMC9324424; doi:10.3390/ijms23147987)
Supplement: Supplementary file 1 [file ijms-23-07987-s001.zip › Supplementary Table S2.pdf]

Supplementary Table S2. Clinico-pathological characteristics of plasma study cohort

	**Indolent group**		**Up-grading group**	
	n=60		*n=20*	
**Variable (at Diagnosis)**	**Median**	**IQR**	**Median**	**IQR**
Age (years)	62.7	58.0-69.0	65.5	61.8-70.5
PSA (ng/mL)	5.65	4.70-6.71	6.05	4.58-6.82
Prostate volume (cm^3)	55.4	37.0-62.3	43.6	33.0-46.3
PSA density (ng/mL/cm^3)	0.11	0.08-0.13	0.15	0.12-0.18
Max PCa length (%)	13.3	5.0-20.0	18.5	5.0-30.0
Positive cores (n)	**n (%)**		**n (%)**	
≤1	46 (76.7)		19 (47.5)	
>1	14 (23.3)		21 (52.5)	
Positive cores (%)				
<10	37 (61.7)		16 (40.0)	
≥10	23 (38.3)		24 (60.0)	

IQR= interquartile range
