# Supplementary material for: Potential of the Stromal Matricellular Protein Periostin as a Biomarker to Improve Risk Assessment in Prostate Cancer
Source: Int J Mol Sci. 2022 Jul 20;23(14):7987. doi: 10.3390/ijms23147987 (PMC9324424; doi:10.3390/ijms23147987)
Supplement: Supplementary file 1 [file ijms-23-07987-s001.zip › Supplementary Table S3.pdf]

Supplementary Table S3. Contribution of each variable of the final model

Variable	Harell C- statistic
**Final model**	0.737
**Without each of the following variables**	
Resection margins	0.654
Periostin	0.663
